# A real-time GPU-accelerated parallelized image processor for large-scale multiplexed fluorescence microscopy data

**DOI:** 10.3389/fimmu.2022.981825

**Published:** 2022-09-23

**Authors:** Guolan Lu, Marc A. Baertsch, John W. Hickey, Yury Goltsev, Andrew J. Rech, Lucas Mani, Erna Forgó, Christina Kong, Sizun Jiang, Garry P. Nolan, Eben L. Rosenthal

**Affiliations:** ^1^ Department of Otolaryngology, Stanford University School of Medicine, Stanford, CA, United States; ^2^ Department of Microbiology and Immunology, Stanford University School of Medicine, Stanford, CA, United States; ^3^ Department of Pathology, Stanford University School of Medicine, Stanford, CA, United States; ^4^ Department of Hematology, Oncology and Rheumatology, Heidelberg University Hospital, Heidelberg, Germany; ^5^ Center for Virology and Vaccine Research, Beth Israel Deaconess Medical Center, Harvard Medical School, Boston, MA, United States; ^6^ Department of Otolaryngology, Vanderbilt University Medical Center, Nashville, CA, United States

**Keywords:** image processing, highly multiplexed imaging, CODEX imaging, image deconvolution, drift compensation, GPU acceleration, parallel computing, big data

## Abstract

Highly multiplexed, single-cell imaging has revolutionized our understanding of spatial cellular interactions associated with health and disease. With ever-increasing numbers of antigens, region sizes, and sample sizes, multiplexed fluorescence imaging experiments routinely produce terabytes of data. Fast and accurate processing of these large-scale, high-dimensional imaging data is essential to ensure reliable segmentation and identification of cell types and for characterization of cellular neighborhoods and inference of mechanistic insights. Here, we describe RAPID, a Real-time, GPU-Accelerated Parallelized Image processing software for large-scale multiplexed fluorescence microscopy Data. RAPID deconvolves large-scale, high-dimensional fluorescence imaging data, stitches and registers images with axial and lateral drift correction, and minimizes tissue autofluorescence such as that introduced by erythrocytes. Incorporation of an open source CUDA-driven, GPU-assisted deconvolution produced results similar to fee-based commercial software. RAPID reduces data processing time and artifacts and improves image contrast and signal-to-noise compared to our previous image processing pipeline, thus providing a useful tool for accurate and robust analysis of large-scale, multiplexed, fluorescence imaging data.

## 1 Introduction

High-dimensional, single-cell imaging yields multiscale biological information from the molecular to the cellular to the tissue level to enable the inference of biological mechanisms and to drive therapeutic and diagnostic development (1). Over the past decade, several highly multiplexed fluorescence imaging techniques, including MxIF, t-CyCIF, and CODEX, have been developed to enable the imaging of over 50 antigens in a single tissue section (2). These multiplexing techniques typically use cyclic immunostaining and imaging to circumvent the spectral limitation of conventional microscopes. Given the complexity and time-consuming nature of these experiments, it is necessary to quickly examine the acquired and processed images from the first few cycles as a data sanity check and to allow optimization of experimental conditions (e.g., exposure time) before additional experiments are performed. Moreover, with high-dimensional imaging datasets on the order of terabytes now routinely generated, efficient and accurate image processing is imperative for reliable and reproducible downstream analysis.

Although different highly multiplexed fluorescence imaging techniques rely on different modes of antibody tagging (e.g., fluorophores, DNA oligonucleotide barcodes) and different tissue handling protocols, they all involve iterative, multicycle image acquisition using conventional fluorescence microscopes. The raw imaging data typically consists of multiple tile scans (i.e., fields of view) over a large tissue region across many cycles with 2 to 4 channel images per tile and multiple z planes per image volume. To view these marker images, it is necessary to generate montages from individual tiles and to align these montages from different cycles to create a high-dimensional imaging dataset also called hyperstack (x, y, channel, cycle).

At this scale, creation of high-dimensional imaging datasets is challenging. For example, microscopes are often affected by out-of-focus light and noise from the light source and the camera, resulting in blurry and noisy images. There is also axial and lateral drift during the cyclic imaging process that can lead to misalignment. In addition, tissue autofluorescence from endogenous fluorophores (i.e., erythrocytes, collagen, elastin) can result in low signal-to-noise ratios in the imaging data, confounding the detection of weakly expressed antigens. To reliably quantify protein markers and assign cell identities in these large-scale imaging datasets, it is important to address all issues associated with the cyclic tissue imaging process.

A number of tools have been developed for processing high-dimensional fluorescence imaging data. One such pipeline, the CODEX Uploader, which is implemented as open-source Java package, was developed in our lab as part of the study describing the first version of CODEX multiplexed imaging (3). An ideologically similar pipeline was implemented in Python as a part of the Cytokit toolbox (4). The CODEX Uploader has a graphical user interface (GUI) that allows users without any programming background to easily process the complex multiplexed imaging data. This pipeline was used in proof-of-principle validations of CODEX fluidics and antibody DNA-barcoding technology for spatial biology discovery (5, 6).

The CODEX Uploader works well for small-scale multiplexed imaging data with single-tile format (~1,000-10,000 images) (5, 6). However, it is slow when handling larger datasets with large tissue regions involving multiple tiles (>100,000 images on the order of terabytes). With these large datasets, the runtime of the CODEX Uploader is on the order of days on server computers (RAM = 256 GB, 2.6 GHz dual-processors). Moreover, axial and lateral drifts are not adequately addressed in the CODEX Uploader. The CODEX Uploader uses volumetric 3D drift compensation, which was designed to maintain an alignment across the full volume of the z-stack (3). This can result in suboptimal maintenance of focal plane within each z-stack. The 3D drift compensation was originally intended to couple with 3D watershed driven cell segmentation, which was proven to perform efficiently in immune tissues tightly packed with uniformly sized nuclei (3). In heterogeneous cancer tissues, we found that single plane-based neural network-driven segmentation algorithms outperform watershed-based segmentation (7). The single plane-based algorithms demand maintenance of focus across cycles in the aligned stacks, which in a number of cases the CODEX Uploader cannot provide. Furthermore, the CODEX Uploader does not adequately suppress tissue autofluorescence from intensely fluorescent tissue components such as erythrocytes, potentially confounding the detection of low abundance antigens. Therefore, computational tools for efficient and accurate processing of large-scale high-dimensional imaging data involving multiple tiles (>100,000 images on the order of terabytes) are needed.

To address this need, we developed a second-generation CODEX data processor with a parallelized GPU-accelerated image processing framework, which we call RAPID for **R**eal-time GPU-**A**ccelerated **P**arallelized **I**mage processor for massive multiplexed fluorescence microscopy **D**ata. We compared RAPID to the CODEX Uploader on multiplexed imaging data of formalin-fixed paraffin-embedded (FFPE) human tissue of pancreatic ductal adenocarcinoma (PDAC) and cervical lymph nodes. Due to the use of parallelized GPU acceleration, processing time with RAPID was 2- to 3-fold faster than that of the CODEX Uploader with significantly improved image quality. By correcting for axial and lateral drifts and minimizing tissue autofluorescence, RAPID also improved image contrast and signal-to-noise and reduced artifacts and high-intensity background signal. RAPID allows the processing of any pre-selected regions or cycles while image acquisition is ongoing, thus enabling the review of processed images shortly after image acquisition. In summary, RAPID (https://github.com/nolanlab/RAPID) is an open-source software that users can debug, modify, and extend to address various image processing tasks.

## 2 Results

### 2.1 RAPID enables faster image processing of CODEX multiplexed imaging data

**Figure 1:**
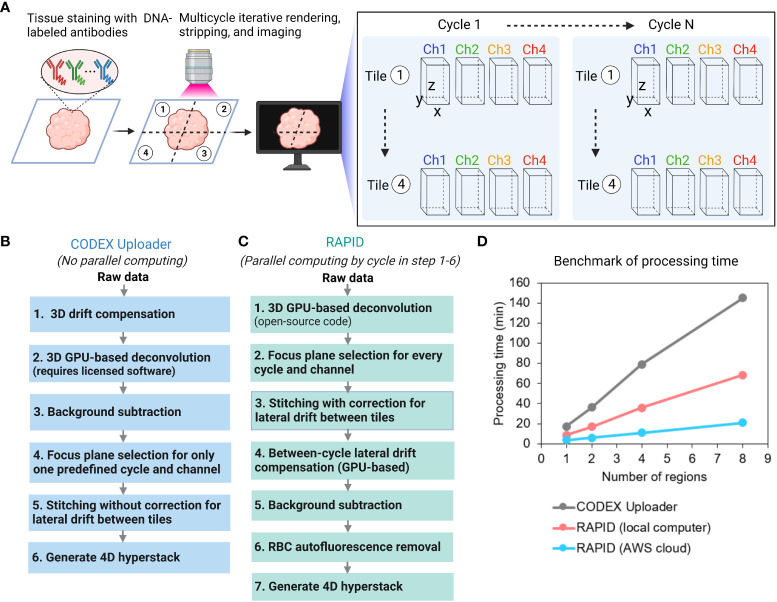
Overview of image processing methods for CODEX multiplexed imaging data. **(A)** CODEX workflow and the structure of a raw imaging dataset. **(B)** Overview of the CODEX Uploader pipeline. **(C)** Overview of the RAPID pipeline. **(D)** Plot of processing time versus number of regions analyzed with the two pipelines on a local system and for the RAPID run on a cloud-based system.

CODEX multiplexed imaging is an iterative process during which a z-stack of one tile is acquired in a given filter channel before switching to the next filter channel and then moving to the next tile until data on the entire tissue region of interest is captured. This process is repeated in multiple cycles with fluorescently labeled oligonucleotides that detect oligonucleotide-coupled primary antibodies (**Figure 1A**). Currently, a typical CODEX multicycle experiment for a 30-core (core diameter = 1 mm) tissue microarray with 53 protein markers involves nine tiles per core, four channel images per tile, and six planes per image volume over 26 cycles and produces over 210,000 images, about a terabyte of data. A single-tile experiment (per tissue sample or coverslip) can be imaged in 2 days, but multi-tile experiments are typically imaged in 4 to 6 days. The CODEX Uploader takes about a day to process one terabyte CODEX dataset.

The CODEX Uploader is limited in several aspects when processing multi-tile CODEX data (**Figure 1B**). First, it only corrects for lateral drift between different imaging cycles within the same tile but it does not account for lateral drift between tiles, leading to misalignment along the edges of tiles in the mosaic. Second, following volumetric 3D drift compensation, it selects the best focus plane for only in one predefined cycle and channel (typically, cycle 1 and the nuclear channel) and then uses the same z-plane for the remaining cycles, which can result in unfocused images in the mosaic and imperfect maintenance of focus. Third, it does not adequately suppress high-intensity autofluorescence (e.g., red blood cells (RBCs)), confounding the detection of low abundance antigens using fluorescence. Finally, the CODEX Uploader runtime scales up considerably with antigen number, tissue area, and sample size, making it impractical to check image quality before all data has been collected. To address these limitations, we developed RAPID, which is parallelized and GPU accelerated. RAPID employs different processing algorithms (**Figure 1C**).

RAPID includes an open-source 3D GPU-based image deconvolution (8) in contrast to the commercial deconvolution software from Microvolution required for use of the CODEX Uploader. RAPID corrects for axial drift by selecting the best focus plane for every channel and every cycle. It then applies the compensation of offsets relative to these planes, preventing unfocused images in the final mosaic. Further, it corrects for lateral drift by computing the spatial shift between two images using Fourier-based approaches. Additionally, it minimizes high-intensity tissue autofluorescence with a customized autofluorescence removal pipeline. **Table 1** summarizes the main differences between the CODEX Uploader and RAPID.

**Table 1:** Comparison between the old and new CODEX image processing pipelines.

	RAPID	CODEX Uploader
Parallel computing	Yes	No
3D GPU-based deconvolution	Yes (open source)	Yes (commercial, >$5k)
Correction for axial drift	Yes	Yes (insufficient)
Correction for between-cycle lateral drift	Yes	Yes
Correction for within-cycle lateral drift	Yes	No
High-intensity autofluorescence removal	Yes	No
Modular	Yes	No
Processing in parallel with CODEX experiment	Yes	No
Graphical user interface	No	Yes

Comparison of RAPID and CODEX Uploader.

RAPID has several distinct advantages (**Figure 1C**). First, RAPID can process any portion of the full dataset as soon as the data are acquired such that the data acquisition and processing is occurring simultaneously which has two important consequences – the image quality can be assessed during data acquisition and reduces time from acquisition and processing from a number of days down to a few hours. Therefore, data quality can be intermittently assessed, allowing the user to terminate data acquisition if needed. Second, GPU-based computation is implemented using the gpuArray object and the gpuArray-enabled MATLAB functions for time-consuming steps such as deconvolution and image registration. Third, RAPID loops through the data cycles using the parfor function in MATLAB to process each cycle independently in parallel on multicore CPUs. The two most time-consuming steps are deconvolution and image stitching. The GPU-acceleration parallel computing used in RAPID reduced the deconvolution time by 13 fold compared to deconvolution without parallelization. Image stitching with correction for lateral shift between tiles was not implemented in the CODEX Uploader. In RAPID, the fast microscopy stitching algorithm MIST is used to estimate the tile positions and parallel computing is employed to create mosaics; this was 6-fold faster than the same steps completed with the conventional ImageJ Grid/Collection plugin.

To benchmark the runtime, both the CODEX Uploader and RAPID were used to process the same multiplexed dataset of eight regions (9 tiles per region and 6 z-planes per tile acquired with 4 wavelength channels over 9 cycles) on the same computer equipped with two low-cost graphics processing units (NVIDIA GeForce GTX 1080). RAPID used 9 CPU cores for parallel computing and had a total runtime two-fold faster than CODEX Uploader (**Figure 1D**). To enable scalable and reproducible workflows, RAPID was deployed on the commercial cloud computing platform Amazon Web Services (AWS). By taking advantage of the high GPU/CPU resources and network bandwidth available on AWS, the analysis was 3-fold faster than on the desktop computer at a cost of approximately $1 per 1000 input tiles (**Figure 1D**). A preconfigured server image is publicly available at https://github.com/nolanlab/RAPID.

### 2.2 RAPID employs open-source GPU-accelerated 3D image deconvolution

Since multiplexed fluorescence imaging data are intrinsically blurred due to the diffraction-limited nature of optical systems, it is necessary to use image deconvolution to partially reverse the image blurring, thus improving image resolution and contrast. The standard Richardson-Lucy deconvolution (RLD) method reduces noise contamination and deburrs images but calculates corrected images iteratively, resulting in high computational burden. The CODEX Uploader utilizes a commercial software that runs a GPU-accelerated RLD algorithm (9). In RAPID, we incorporated a new, open-source method for rapid GPU-based image deconvolution that only requires a single iteration and is faster than the classic RLD method (8).

**Figure 2:**
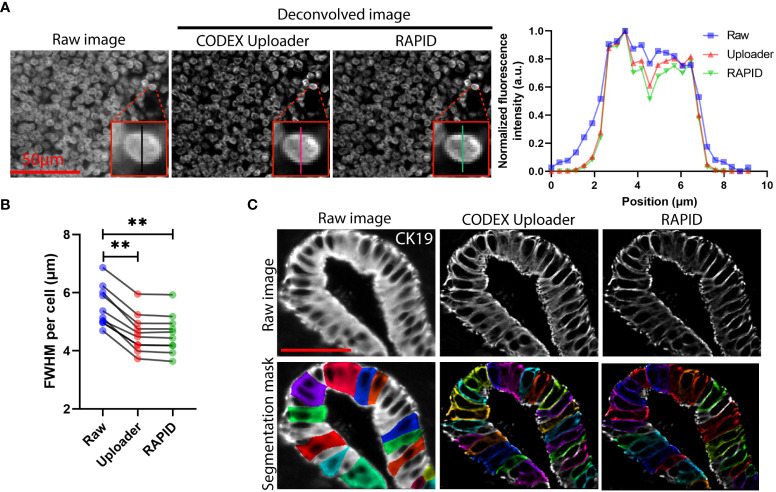
3D image deconvolution improves image contrast in CODEX data. **(A)** Representative raw and deconvolved images of a cervical lymph node, and plot of normalized fluorescence intensity versus lateral position of a selected cell corresponding to the raw and deconvolved images. **(B)** Quantification of the FWHM for signals cross ten randomly selected cells. **(C)** Representative raw and deconvolved images of cytokeratin 19-stained pancreatic ducts and corresponding predicted whole-cell segmentation mask. Wilcoxon matched-pairs signed rank test was used in (**B**). **P < 0.01.

We demonstrated that the open-source algorithm reduced blur and improved resolution of CODEX images as effectively as the commercial software utilized by the CODEX Uploader (**Figure 2A**). The full-width at half maximum of the nuclear fluorescence intensity for 10 randomly selected cells in RAPID processed images was consistently lower than the raw data and was similar to CODEX Uploader (**Figure 2B**). Furthermore, use of the open-source deconvolution algorithm reduced blur and increased image contrast enabling more accurate cell segmentation than achieved without the deconvolution (**Figure 2C**).

### 2.3 RAPID corrects for both lateral and axial drifts and removes image distortion

**Figure 3:**
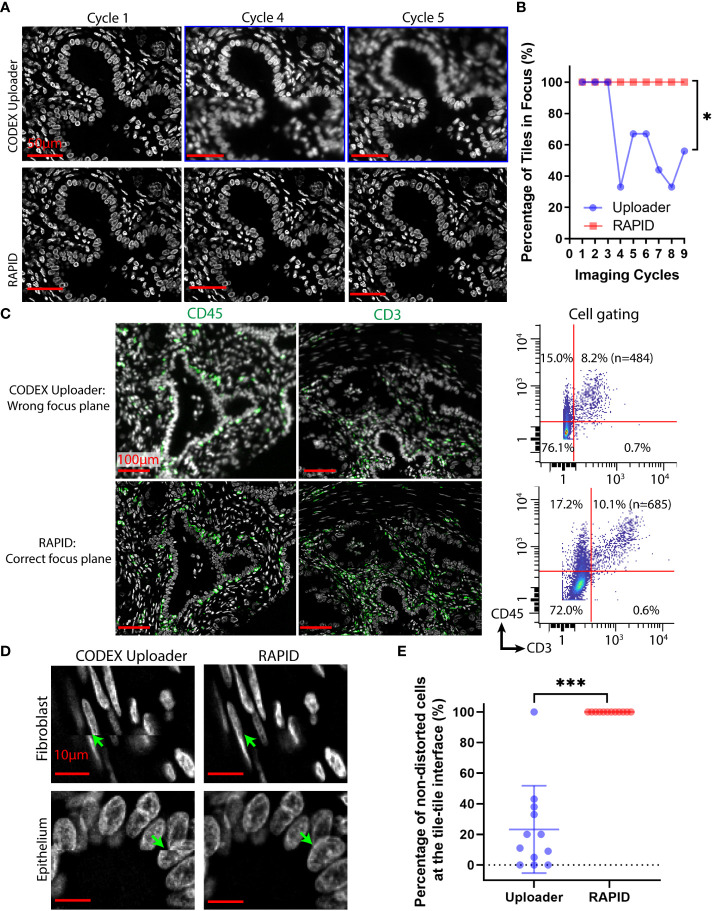
RAPID corrects axial and lateral drifts in CODEX multiplexed data. **(A)** Representative CODEX nuclear images from cycles 1, 4, and 5 of a PDAC tissue section processed by the CODEX Uploader and by RAPID. **(B)** Percentage of in-focus tiles across nine tiles and nine imaging cycles for one example tissue region. **(C)** Representative CODEX images of PDAC tissue section stained for CD45 and CD3 processed by the two pipelines, and the corresponding cell gating using CD3 and CD45 protein expression. **(D)** Representative images of fibroblasts and epithelium processed by the two pipelines. **(E)** Percentage of the non-distorted cells across the twelve tile-tile interfaces of an example tissue region. Wilcoxon matched-pairs signed rank test was used in **(B, E)**. *P < 0.05, ***P < 0.001.

To evaluate the impact of the drifting issues, the CODEX Uploader was compared against RAPID on a 3×3 tile CODEX dataset of human PDAC tissue. Although the best focus plane is typically captured within a z-stack, the position of this focal plane within the z-stack can change over time due to axial drifts. The image data processed by the CODEX Uploader sometimes contains out-of-focus images in imaging cycles beyond the first cycle (**Figures 3A-C**). For example, in a 9-cycle 9-tile multicycle dataset, six of all the cycles contain out-of-focus images after processing by the CODEX Uploader (**Figures 3A, B** **)**. In contrast, RAPID selects the best focus plane for every tile across every channel and cycle, thereby avoiding unfocused images in later cycles (**Figures 3A, B** **)**. The focus correction for CD45 and CD3 cycles performed in RAPID resulted in quantification of 2% more T cells in multiplexed imaging data than identified with the CODEX Uploader (**Figure 3C**).

Lateral drift involves spatial shifts between tiles in sequential imaging cycles and within the same imaging cycle. The CODEX Uploader compensates for lateral drift in different imaging cycles but ignores within-cycle drift during tile scanning and generates a mosaic after cropping the overlapping region between tiles. As a result, the image montage generated by the CODEX Uploader contains distorted cells at the interface of two adjacent tiles (**Figures 3D, E** **)**. In contrast, RAPID uses phase correlation-based image registration to correct for both the axial and lateral spatial shifts, thus producing focused tiles without axial misalignment between tiles, improving cell identification (**Figures 3D, E** **)**.

### 2.4 RAPID removes high-intensity fluorescent artifacts and improves weak marker detection

Tissue autofluorescence can interfere with the immuno- fluorescence detection of antigens, especially for low abundance markers in Alexa Fluor 488 and Cy3 channels. The sources of autofluorescence include endogenous tissue components such as RBCs, collagen, elastin, and lipofuscin and can also result from tissue-processing techniques such as formalin fixation (10). Although chemical treatments can quench tissue autofluorescence, these reagents may not compatible with dimethyl sulfoxide used in the CODEX imaging protocols, and currently there is no standardized method for tissue treatment that minimizes autofluorescence. Therefore, image processing methods are frequently used to reduce the impact of fluorescent artifacts. For example, background images can be generated using a rolling ball algorithm (with a 50-pixel radius) and then subtracted from marker images for CyCIF multiplexed imaging data (11). For CODEX imaging, it is common practice to acquire background autofluorescence (blank) images across all the imaging channels before the rendering step at the beginning of, during, or after the multicycle imaging process. In analysis with the CODEX Uploader, images from the first blank cycle are typically subtracted from the marker images to alleviate the impact of tissue autofluorescence.

**Figure 4:**
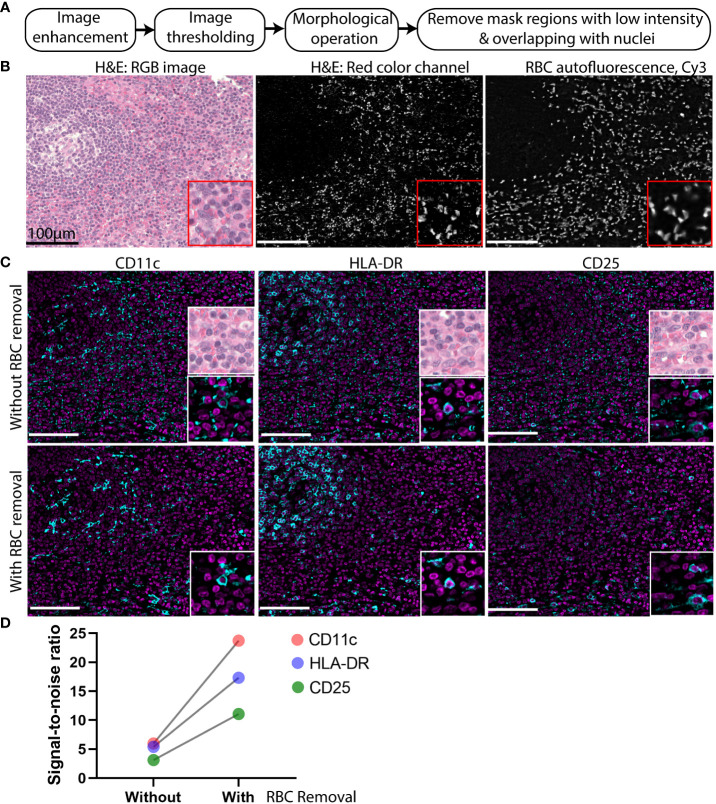
High intensity autofluorescence removal improves signal to noise of CODEX multiplexed imaging data. **(A)** Flowchart of the high intensity autofluorescence removal method. **(B)** Image of an H&E-stained cervical lymph node. Left: RBCs manifest as bright pink patches widely dispersed among the cells (nuclei indicated by purple pixels). Middle: the pink color image was digitally separated from the H&E image shown in grayscale. Right: the blank CODEX image from Cy3 channel of the same tissue section. **(C)** Representative images of weak markers CD11C, HLA-DR, and CD25 (cyan) overlaid with nuclear stain (magenta) without and with application of the RBC removal algorithm. **(D)** Signal-to-noise ratio of three markers CD11c, HLA-DR, and CD25 with and without RBC removal.

Although the background subtraction method generates acceptable results for most endogenous tissue components such as collagen and elastin, it is unreliable for objects with high intensity autofluorescence such as that from RBCs. For RBCs, differences in intensities between imaging cycles or linear-scaling at different exposure times can result in persistence of a considerable amount of autofluorescence after background subtraction. RBCs are commonly detected in the extracellular spaces, likely from bleeding into the tissue during surgical removal of specimens. These RBCs can confound the detection of low abundance markers in nearby cells. Therefore, we designed an image processing strategy that first normalizes and enhances the blank images, next identifies the high intensity pixels by global thresholding and removal of small objects (artifacts), and then applies two additional thresholding steps to remove low intensity objects and false positive cells (**Figure 4A**).

To confirm that RBCs are a frequent source of high-intensity background objects, a hematoxylin and eosin (H&E) stain of a lymph node was analyzed. To digitally isolate the RBCs in the H&E image, color deconvolution (12) was applied to separate the RBCs from the nuclear stain (**Figure 4B**). The RBC areas isolated from the H&E images co-localized well with the brightly fluorescent regions identified from the blank CODEX image (**Figure 4B**).

To demonstrate the importance of RBC removal for accurate detection of low-abundance markers, the standard background subtraction method was compared against the method used in RAPID by application to CODEX images of a cervical lymph node (**Figure 4C**). Visualization of weakly fluorescent markers such as CD11c, HLA-DR, and CD25 were hampered due to persistent autofluorescence artifacts after the standard subtraction of a blank image (**Figure 4C**). After applying the additional RBC removal step used in RAPID, we saw improved visualization of the weak markers (**Figure 4C**) and high signal-to-noise ratio in the images with RBC removal (**Figure 4D**).

### 2.5 RAPID improves down-stream cell-type identification

To demonstrate the impact of different image processing methods on downstream analysis, processing results of CODEX images of a cervical lymph node tissue, which contained intense autofluorescence from RBCs, with the CODEX Uploader were compared against those from RAPID. Following image processing using the two pipelines, cell segmentation, protein quantification, unsupervised clustering, and cell-type annotation were performed on over 8,000 cells from a CODEX image hyperstack with a single tile and 21 protein markers. These markers allowed identification of lymphoid lineage cells.

**Figure 5:**
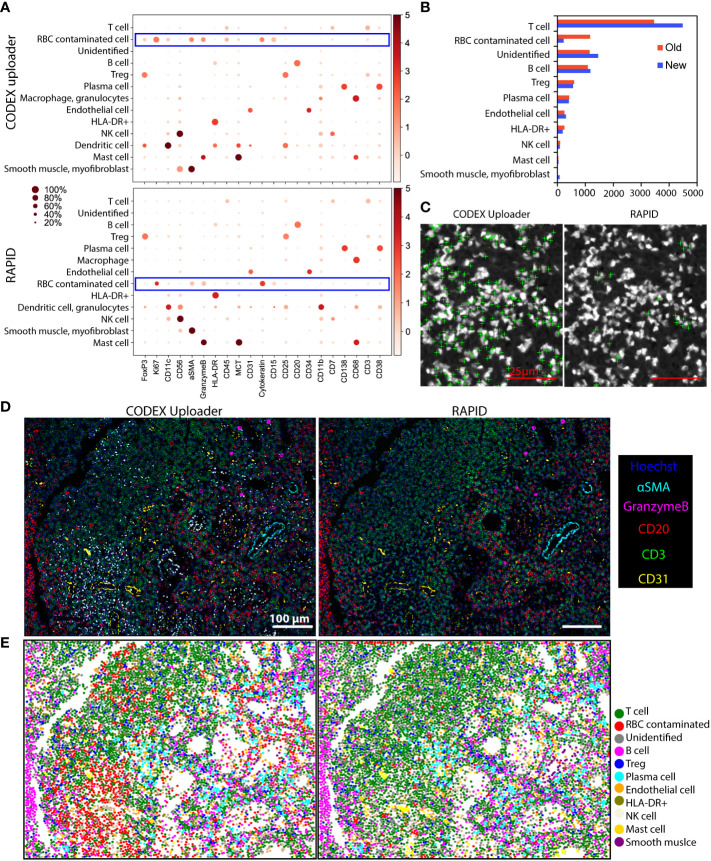
RAPID improves down-stream cell-type identification. **(A)** Unsupervised clustering and cell-type annotation of CODEX data processed using the CODEX Uploader (total number of cells = 8967) and RAPID (total number of cells = 9597). **(B)** Bar graphs of the numbers of annotated cells identified by the two pipelines. **(C)** Overlays of a blank image (Cy3 channel) with cell centroids (green crosses) assigned to the RBC-contaminated cell clusters after processing with the two pipelines. **(D)** Overlays of Hoechst nuclear stain with CD3, CD20, CD31, αSMA, and Granzyme B stainings of images processed with CODEX Uploader and with RAPID. Noise from RBC autofluorescence is indicated by white (due to overlay of all colors). **(E)** Scatter plots of x/y coordinates of cell centroids showing the spatial distribution of the annotated clusters in the tissue. Centroids of cells from the RBC contaminated cluster (orange) co-localize with white spots in images in panel **(D)**.

Both pipelines identified the same cell types, but RAPID assigned fewer cells to an inconclusive, RBC-contaminated cluster (**Figure 5A**) and identified nearly 30% more T cells (**Figure 5B**). An overlay of RBC-contaminated cell clusters on the RBC autofluorescence image using the CODEX Uploader revealed that most cells in this cluster were close to strongly fluorescent artifacts from RBCs; however, significantly fewer cells were assigned to this cluster after RAPID processing due to the autofluorescence removal step (**Figure 5C**). This indicates that RAPID enables more accurate identification of cell types affected by strongly autofluorescent artifacts from tissue components such as RBCs.

To further demonstrate the value of RBC autofluorescence removal, composite images of five markers (Hoechst, CD20, CD3, αSMA, and granzyme B) were generated after processing with the two pipelines. Since these are mutually exclusive markers, overlapping areas, which manifested as the white areas in the images, were indicative of RBC autofluorescence (**Figure 5D**). The RBC removal step in RAPID dramatically reduced autofluorescence and resulted in identification of a higher number of total cells than identified when the images were processed with the CODEX Uploader (**Figure 5E**). In addition, the CODEX Uploader applied a scaling factor to the deconvolved images, thereby reducing the overall fluorescence intensity of the nuclear images. In RAPID there is no scaling step, leading to an overall higher fluorescence intensity and improved detection of cells marked by low-intensity signal.

## 3 Discussion

Highly multiplexed, single-cell fluorescence imaging methods such as CODEX have revolutionized our understanding of the spatial architecture and multicellular interactions that occur in healthy and diseased tissues. To fully leverage the power of these multiplexed imaging technologies, it is important to efficiently process the high-dimensional imaging data to allow for reliable downstream analysis and extraction of meaningful biological information. As these multiplexed techniques are advanced to allow imaging of more markers, larger tissue regions, and more samples, the amount of imaging data also drastically increases. Big-data-based analytics are expected to drive the next wave of therapeutic and diagnostic development; however, the development of new algorithms and computational tools to process these huge datasets have lagged behind technology development.

A few CODEX image preprocessing methods have been reported in the literature. Co-developed with the CODEX imaging technology, the CODEX Uploader was the first-generation CODEX image processor and it was originally optimized based on performance in the multilayered slices of frozen murine immune tissues where the volumetric watershed segmentation showed robust and reproducible performance. Yet, for large-scale high-dimensional data (>100,000 images), the processing time of the CODEX Uploader is slow (on the order of days). In addition, CODEX Uploader sometimes leads to suboptimal maintenance of focal plane across large set of tiles and it does not perform lateral drift compensation to reduce the artifacts on the borders between the tiles. Cytokit (4) implemented an image processing pipeline that is essentially the same as the original CODEX Uploader. Like CODEX Uploader, Cytokit lacks lateral drift correction and removal of strong tissue autofluorescence. Cytokit used the Richardson Lucy algorithm for image deconvolution, which is slow for the large-scale dataset. In contrast, RAPID parallelized a GPU-accelerated deconvolution algorithm (8), which was much faster and produced similar or better image quality than the Richardson Lucy algorithm. MCMICRO (13) is another toolbox for processing and analyzing multiplexed imaging data, but it lacks 3D deconvolution that is beneficial for multiplexed imaging data with multiple z-planes like CODEX.

To address these issues, we developed a parallelized GPU-accelerated image process pipeline called RAPID for fast and accurate processing of large-scale high-dimensional data. RAPID reduced the runtime by 2~3 fold relative to CODEX Uploader and improved image quality by reducing noise and artifacts. To enable scalable and reproducible analysis for CODEX users, we have made a server image of RAPID available for use on AWS. This cloud computing option enables users to begin analysis with little need for configuration. The server-based version is three times faster than the local version at a cost of approximately $1 per 1000 input tiles. As RAPID can be set up to process any number of regions and cycles, it can run in the background during image acquisition with processing finished almost immediately following the completion of the full experiment. As a result image acquisition can be terminated if there are quality issues but also considerably shortens the time for downstream big data analysis.

RAPID also improves image quality and downstream data analysis by introducing two new components (1): accurate correction for axial and lateral drifts, which are inevitable during extended cyclic imaging process, and (2) effective detection and removal of intense tissue autofluorescence artifacts. The drift correction implemented in RAPID removes cellular distortion in the final mosaic and leads to more accurate protein quantification. Removal of autofluorescence reduces noise interference with protein markers, especially those present in low abundance. In addition, we demonstrated that the freely available open-source deconvolution algorithm (8) used in RAPID produces improved image contrast enabling accurate whole-cell segmentation and quantification that is similar to what was obtained with the commercial deconvolution software in the CODEX Uploader. The analyses reported here highlight the importance of using deconvolution to partially reverse the intrinsic blur and noise in the multiplexed fluorescence images. In addition, RAPID processing of images with high-intensity background contamination resulted in an improved signal-to-noise compared to the images processed with the CODEX Uploader. Further, RAPID improved downstream detection and segmentation of low-intensity cells as well as cell-type annotation.

Although we only demonstrate the application of RAPID for CODEX data, the same pipeline can be broadly applicable to other multiplexed fluorescence imaging platforms that depend on repeated fluorescence microscopic imaging. RAPID is open-source and modular, which allows addition or removal of any image processing steps. The 3D deconvolution can be used for any fluorescence microscopic data with multiple z-plane images. All the other modules apply to any multiplexed fluorescence imaging data. To use RAPID for different multiplexed imaging techniques, it may require changes to the directory and file naming of the raw data and imaging parameters used in the processing algorithm (e.g. exposure time, image resolution, etc.). We have specified instructions on the GitHub page. In the future, a user-friendly GUI will be added and batch normalization methods could be included to take into consideration the variations caused by factors such as different microscopes or users rather than biology.

## 4 Materials and methods

### 4.1 CODEX multiplexed tissue staining and imaging

Tissue microarrays of human pancreatic ductal adenocarcinoma (core size: 1 mm) and cervical lymph nodes (core size: 1 mm) were sectioned at 4 μm and mounted onto Vectabond™-treated glass coverslips (25 mm × 25 mm). The patient tissues used for this study were fully anonymized, and use was approved by the Stanford University Institutional Review Board. Written informed consent was obtained from all patients. Commercially available, purified, carrier-free anti-human antibodies (**Table S1**) were conjugated to maleimide-modified DNA oligonucleotides and titrated on the tissue of interest following previously published protocols (5, 14, 15). All the antibodies used were previously validated for use in CODEX multicycle imaging (5). The multicycle image acquisition was conducted using Akoya’s CODEX instrument connected to a Keyence BZ-X700 microscope configured with four fluorescent channels (DAPI, FITC, Cy3, Cy5) and a 20x objective. A 3×3 tiled acquisition (6 z-planes) and 1×1 tile (17 or 29 z-planes) were used for PDAC and the lymph node sections, respectively. The CODEX dataset acquired on a Keyence microscope has the following parameters (1): tile: a single field of view (2); region: tissue region of interest composed of single tile or multiple tiles collected as rectangular grids (3); z: the number of z planes acquired in the axial direction that includes the best focus plane and additional planes above and below the best focus plane (4); channel: images acquired through optical filters for imaging DAPI, Alexa Fluor 488, Cy3, and Cy5 (5); cycle: group of two to four imaging channels acquired between oligonucleotide-fluorophore reactions.

### 4.2 RAPID

RAPID is fully implemented in MATLAB (version R2020a) and requires pre-installation of the MIJ toolbox to allow ImageJ and Fiji to run within MATLAB (16). The source code and user instructions were released under the GPL License at GitHub website (https://github.com/nolanlab/RAPID). RAPID consists of four main components described in the subsections below:

#### 4.2.1 GPU-accelerated 3D image deconvolution

In the CODEX multicycle imaging process, a z-stack of a tissue sample is acquired from a series of 2D images by focusing the fluorescence microscope at different planes. Each 2D image acquired by the fluorescence microscope contains both in-focus and out-of-focus information and is affected by noise from light source and camera, leading to blur that reduces lateral resolution. This blur is usually modeled by the point-spread function (PSF), which is the 3D impulse response of a fluorescence microscope system. Image deconvolution aims to computationally estimate the underlying sample from noisy, blurred images. The CODEX Uploader (https://github.com/nolanlab/CODEX) utilized a commercial deconvolution software, which implemented the standard RLD algorithm as the Microvolution ImageJ plugin (9). The following Microvolution settings were used: iteration number = 25, deconvolution mode = “vectorial”, numerical aperture = 0.75, per_pixel_XY_resolution = 377.442, z_pitch = 1500.0, num_z_planes = 6 or 17 or 29, emission_wavelengths = [425, 525, 595, 670]. In RAPID, a rapid image deconvolution algorithm (8) was incorporated. First, the PSF of the microscope for each imaging filter (DAPI, Alexa Fluor 488, Cy3, Cy5) was estimated using an ImageJ plugin PSF generator (http://bigwww.epfl.ch/algorithms/psfgenerator/) with the following parameters: Optical model = Born and Wolf, wavelength = 425 or 525 or 595 or 670], numerical aperture = 0.75, Pixel size XY = 377.44 nm, Z-step = 1,500 nm, Size XYZ = 255×255×number of z planes (6 or 17 or 29), Display = Grays. Next, a Wiener-Butterworth filter was used as the unmatched back projector to accelerate the RLD algorithm. This produces deconvolved images with similar or better image quality than the traditional RLD algorithm (8). Three parameters are used to fully specify the Wiener-Butterworth filter (1): alpha: a small value used to ensure that inverting the forward projector does not result in division by zero (good results are obtained with alpha in the range of 0.001~0.005) (2); beta: spectral amplitude at the cutoff frequency or resolution limit (set at a small value to suppress spatial frequencies beyond the resolution limit); and (3) n: the filter order used to set the transition slope at the cutoff frequency (i.e., the highest possible spatial frequency passed by the microscope). The parameters for RAPID were set as follows: iteration = 1, alpha = 0.05, beta = 0.1, n = 20.

#### 4.2.2 Axial focus drift correction

The CODEX image data consists of a series of z-stacks in two to four filter channels across many imaging cycles. Each z-stack contains the focal plane and planes above and below the focal plane. The best focus plane may drift within the z stack over the course of iterative imaging acquisition due to varying tissue thickness or mechanical or thermal factors. To select the focal plane from the z-stacks, we incorporated multiple focus measure operators (17, 18) into the new pipeline. These focus metrics assume that the focused images have more sharp edges or variations or textures than blurred ones. In RAPID, the best focus planes were selected per tile per channel per cycle, accounting for the temporal and spatial shifts of the focus planes.

#### 4.2.3 Lateral drift compensation

Lateral drift in the CODEX data results from spatial shift of tiles across different imaging cycles with reference to cycle 1 and within the same imaging cycle with reference to adjacent tiles. To correct for the between-cycle drift, a phase-correlation-based method (19) is used in RAPID to estimate geometric transformation (translation, rotation, and scaling) that aligns different image cycles. For the reference images, the first cycle and nuclear channel were set as the default (can be changed by users). The geometric transformation of the reference image was subsequently applied to the other image channels and cycles. To correct for the within-cycle drift, we adopted the MIST algorithm (20), which uses phase correlation to compute translations between adjacent tiles and then estimates a mechanical stage model from the computed pair-wise translations. The estimated positions of tiles were used to re-organize individual tiles in 2D space, and linear blending was applied to each tile to compensate for shading differences in overlapping areas between adjacent tiles to generate the final stitched image.

#### 4.2.4 High-intensity autofluorescence detection and removal

High-intensity autofluorescence in FFPE tissue was most prominent in Alex Fluor 488 and Cy3 channels. Here, we chose to use the Cy3 channel image from the first blank or background cycle to identify affected pixels. First, the raw image data was enhanced by min-max normalization followed by contrast-limited adaptive histogram equalization (21). Next, image pixels with high autofluorescence intensities were identified using multilevel image thresholding based on Otsu’s method (22), and a binary mask (1 = bright pixels; 0 = dim pixels) was generated. To refine the mask, small objects with fewer than 10 pixels were considered as noise and removed. To avoid the confounding effect of one or a few very bright pixels, the mean fluorescence intensity of all pixels within individual objects of the remaining mask were calculated, and objects with low mean fluorescence intensity (~20% of the highest intensity level 65535) were removed. In addition, some nucleated cells were also found to be highly autofluorescent. Therefore, objects overlapping with nuclear staining were removed to prevent loss of nucleated cells. Finally, the remaining mask objects were set to zero in the background-subtracted images across all affected imaging channels and cycles.

### 4.3 Cell segmentation and cell-type annotation

To evaluate the accuracy of whole-cell segmentation with and without image deconvolution, the cellPose algorithm (23) was used to segment cells from CODEX images of cytokeratin staining with an average cell diameter of 45 µm. To assess how RAPID processing influenced downstream analysis such as cell-type annotation, a pre-trained mask region-convolutional neural network (R-CNN) was used to segment the nuclei from CODEX images processed by CODEX Uploader and by RAPID. The nuclear masks were morphologically dilated by three pixels to quantify the mean fluorescence intensity of each protein marker. The signal spill-over was compensated for as described (3). After the R-CNN-based cell segmentation, a single csv file was generated for each tissue region of interest that included cell ID, region ID, tile number, the (X, Y, Z) coordinates of the cell centroids, cell size, and the mean fluorescence intensity of each protein marker per cell. Next, Leiden-based clustering was performed with the single-cell protein quantification data from the csv files using the scanpy Python package (24). Finally, the Leiden clusters were visualized in heat maps of dot plots and spatially mapped back to the CODEX multicycle imaging data to allow for manual cell annotations.

## Data availability statement

The original contributions presented in the study are included in the article/**Supplementary Material**. Further inquiries can be directed to the corresponding authors.

## Ethics statement

The studies involving human participants were reviewed and approved by Stanford University Institutional Review Board. Written informed consent for participation was not required for this study in accordance with the national legislation and the institutional requirements.

## Author contributions

GL designed and implemented the algorithm, conjugated antibodies, performed experiments, analyzed and interpreted the data, and wrote the manuscript. MB validated the algorithm, conjugated antibodies, performed experiments, analyzed and interpreted the data, and revised the manuscript. JH conjugated antibodies, analyzed and interpreted the data, and revised the manuscript. YG assisted with issues related to CODEX Uploader, interpreted the data, and revised the manuscript. AR added cloud-based computing and automatic parameter input options, interpreted the data, and revised the manuscript. LM interpreted the data and revised the manuscript. EF and CK constructed tissue microarrays and revised the manuscript. SJ interpreted the data and revised the manuscript. GPN and ER interpreted the data, revised the manuscript, and supervised the study. All authors contributed to the article and approved the submitted version.

## Funding

This work was supported by the U.S. National Institutes of Health (5R01CA238686-02, 5R01CA239257-03, 5U54CA20997103), and Bill & Melinda Gates Foundation INV-002704 (SJ & GPN). GL was supported by the Stanford Molecular Imaging Scholars (SMIS) Program (NIH T32CA118681), the 2021 Stanford Cancer Institute (SCI) Innovation Award, the Stanford Translational Research and Applied Medicine grant, and the National Cancer Institute (NIH K99CA267171). ﻿MB was funded by a Career Development Award of the International Myeloma Society. JH was supported by an NIH T32 Fellowship (T32CA196585), and an American Cancer Society - Roaring Fork Valley Postdoctoral Fellowship (PF-20-032-01-CSM).

## Acknowledgments

The authors thank members of the Nolan lab and the Rosenthal lab for helpful discussion, especially Yunhao Bai and Bokai Zhu. The authors also thank Gustavo Vazquez and Chiara Caraccio for maintaining CODEX reagents in the Nolan lab, Min Guo from NIH for his help with using the rapid image deconvolution method, Shirley Kwok from the Department of Pathology at Stanford Hospital for making tissue microarrays, and Pauline Chu from the Department of Pathology for preparing tissue sections on coverslips. **Figure 1** was created with BioRender.com.

## Conflict of interest

Author GPN received research support from Pfizer, Vaxart, Celgene, and Juno Therapeutics during the course of this work. Authors YG and GPN are inventors on US patent 9909167, granted to Stanford University, that covers some aspects of the technology described in this paper. Authors YG and GPN have equity in and/or are scientific advisory board members of Akoya Biosciences, Inc.

The remaining authors declare that the research was conducted in the absence of any commercial or financial relationships that could be construed as a potential conflict of interest

## Publisher’s note

All claims expressed in this article are solely those of the authors and do not necessarily represent those of their affiliated organizations, or those of the publisher, the editors and the reviewers. Any product that may be evaluated in this article, or claim that may be made by its manufacturer, is not guaranteed or endorsed by the publisher.
